# Exploring the nursing student experience at a remote Australian university campus: a qualitative study

**DOI:** 10.1186/s12912-022-00996-x

**Published:** 2022-08-02

**Authors:** Catherine Hays, Susan Devine, Beverley D. Glass

**Affiliations:** 1 Centre for Rural and Remote Health, James Cook University, Mount Isa, Queensland Australia; 2 College of Public Health, Medical and Veterinary Sciences, James Cook University, Townsville, Queensland Australia; 3 College of Medicine and Dentistry, James Cook University, Townsville, Queensland Australia

**Keywords:** Education, Health workforce, Rural health, Rural nursing, Nursing students

## Abstract

**Background:**

Nurses constitute most of the rural and remote Australian health workforce, however staff shortages in these regions are common. Rural exposure, association, and undertaking rural clinical placements can influence health students’ decision to work rurally after graduation, however attending university in rural and remote regions has been shown to be a great contributor. An improved understanding of these nursing students’ experiences may inform changes to teaching and support strategies for these students, which in turn could improve their retention and completion rates, contributing to a more sustainable rural and remote Australian nursing workforce. This study aimed to explore and describe students’ experiences of studying nursing in the context of a satellite university campus located in a remote town, with a focus on education delivery methods, staff, support, student services, and barriers and enablers to successful study.

**Methodology:**

Nine students participated in this qualitative descriptive study. Semi-structured interviews were undertaken, allowing participants to reflect on their experiences as nursing students in the context of a geographically remote satellite university campus. The resulting data were grouped into common themes and summarised.

**Results:**

Students were generally positive regarding lectures delivered by videoconference or recorded lectures, as they allowed for greater flexibility which accommodated their busy personal lives. Face-to-face teaching was especially valuable, and students were particularly positive about their small cohort size, which enabled the creation of strong, supportive relationships between students, their cohort, and teaching and support staff. However, barriers related to student demographics and some difficulties with course engagement and campus staffing were experienced.

**Conclusions:**

The experiences of nursing students at remote university campuses are different from those experienced by traditional, metropolitan university students. Although these nursing students face additional barriers unique to the remote campus context, they benefit from a range of enabling factors, including their close relationships with other students, staff, family, and their local community.

## Background

Rural and remote Australian populations are at higher risk of chronic disease, mortality, and total disease burden than their regional and metropolitan counterparts. These poor health outcomes are affected by reduced access to primary and specialist healthcare services [1]. Nurses, the backbone of the rural and remote Australian health workforce, account for 68% of registered health professionals in remote and very remote regions [2]. However, nursing staff shortages in these areas are common, as a result of high staff turnover rates, reliance on short-term agency staff, and poor recruitment and retention [2, 3]. These factors contribute to poorer continuity of care and have resulted in nursing staff safety concerns and poorer health outcomes for rural and remote communities [3, 4].

It is well recognised that rural exposure and associations, including rural clinical placements, a rural background, or attending a rural university campus influence health students’ intention to work rurally following graduation [5–7]. The importance of locally based rural and remote nurses has been highlighted during the COVID-19 pandemic, where essential short-term contract and agency nurses travelling from interstate or New Zealand were unable to access vulnerable remote Aboriginal and Torres Strait Islander communities due to restrictions [8, 9].

Studies have found that students who attend satellite university campuses (campuses located remotely from the main university campus, also known as regional or branch campuses) in rural and remote regions are more likely to choose to practice in regional, rural and remote locations following graduation: Playford et al. [6] reported that 50% of rural campus nursing graduates worked in rural areas following graduation compared to 25% of urban graduates; Gum [10] stated that eight out of eleven graduates of a rural nursing campus were retained in the region as registered nurses; and in a study regarding career aspirations by Birks et al. [11], it was found that students from non-metropolitan areas were more likely to choose non-metropolitan areas as their intended location of practice. Rural campus graduates are also more likely to be ‘rural ready’, due to increased familiarity with the rural context [6]. While remote campus students face a number of unique barriers to study, including support from the main campus, negative experiences of education delivery methods such as videoconference, and reduced access to resources including library and computer facilities, they also benefit from a number of enablers, including avoidance of the social, emotional, and financial impact of leaving friends and family to relocate for university [6, 10, 12]. Family, friends and community provide study support, child minding and emotional support to students, supporting students to study [10, 13, 14].

A recent review of the literature identified a lack of current qualitative research regarding experiences of students located at rural and remote satellite nursing campuses, particularly from the students’ perspective [15]. A deeper understanding of these students’ experiences may inform changes to course delivery, staffing, resources, and support strategies, leading to better retention and course completion rates. This in turn may contribute to the sustainability of the rural and remote Australian nursing workforce, resulting in better access for rural and remote populations.

## Methods

### Aims

This study aimed to explore and describe nursing students’ lived experiences of studying at a geographically remote university campus and to understand *what it means to be a nursing student at a university campus located in a remote Australian town*.

This qualitative descriptive study, influenced by van Manen’s hermeneutic phenomenology, was undertaken to gain an understanding of the participants’ daily life experiences [16–18]. The methodology was chosen due to its emphasis of the importance of context, and the ways in which it can influence a person’s experiences [19].

### Study setting

This study examines the experiences of students enrolled in a regional university that offers the Bachelor of Nursing Science (BNSc) program across four sites in Queensland, Australia. The geographically remote satellite campus is attached to a University Department of Rural Health (UDRH) which facilitates and supports health student clinical placements, located approximately 900 km from the main university campus in a town with a Modified Monash Model rurality classification of MM6 (remote community) [20]. Students enrolled in the BNSc undertake lectures, group tutorials and practical classes at the campus. Lecture delivery methods vary depending on the subject and teaching staff availability; however, they are usually transmitted live via videoconference from the main campus, and/or recorded and posted online for the students to view at any time. Group tutorial and practical classes are mostly delivered face-to-face by local teaching staff. Practical classes take place at the remote campus in simulated ward facilities. While most practical classes are undertaken locally, human bioscience laboratories are conducted at the main university campus as a one-week residential block in first year due to the specialised equipment required for these sessions. The campus has one full-time nursing lecturer, who provides teaching and support to all nursing students, while other sessional tutors are employed from the local hospital. The UDRH also provides students access to a clinical library, a computer laboratory, and Wi-Fi for students. Aboriginal and Torres Strait Islander students can also access additional support, including Indigenous Tutorial Assistance and Academic Support Advisors; and support from the Head of Indigenous Health employed at the UDRH.

### Sampling and recruitment

Participants were recruited through purposive sampling of undergraduate nursing students enrolled at the remote satellite university campus. The researchers invited all internal and/or mixed-mode students, who had completed at least two semesters of study (23 students) at the campus, to participate in an interview via email.

### Data collection

Data collection took place between September and November 2020. The researchers developed the interview framework following a review of the literature to investigate education and support strategies, and barriers and enablers to successful study at rural and remote satellite nursing campuses [15]. After an exploratory pilot interview, the questions were refined for the current study. The first author, a female research assistant employed at the University Department of Rural Health undertaking post-graduate education by distance, conducted individual interviews. The interviewer was known to some participants due to the nature of both a small campus and a remote community, however the potential for any prior relationship to influence participant response was considered minimal as the researcher was not involved with nursing student education. The remaining authors, both experienced qualitative researchers and tertiary educators, contributed to project development, review and supervision. Individual face-to-face interviews were undertaken at the remote campus in a non-classroom setting whenever possible; with Zoom or telephone interviews offered as an alternative option. Interviews began with the broad question: “could you tell me about what it’s like to be a student at a remote campus?” which allowed participants to discuss significant factors and experiences that were referred to throughout the interview and influenced probing questions. Participant demographic information was also collected and de-identified. Nine students participated in the study. Interview duration ranged from approximately 22 to 52 minutes, averaging approximately 37 minutes. In preparation for data analysis, the interviews were recorded and transcribed verbatim.

### Data analysis

Transcripts were checked for accuracy and then de-identified. Interview audio tapes were listened to multiple times and transcripts read and re-read to elicit *what statement(s) or phrases seemed particularly essential or revealing about the experience being described* [18]. The data were then organised and labelled according to common themes that were identified during analysis [21]. Qualitative data analysis software NVivo 12 (NVivo qualitative data analysis software; QSR International Pty Ltd. Version 12, 2018) was utilised to manually highlight relevant statements, and assign and manage themes. Two participants were then invited to view and reflect on quotes and draft summaries, to offer feedback and validate the interpretation of the data [18].

### Ethical approval

James Cook University Human Research Ethics Committee granted ethical approval (H8203) for the study. Participants were provided with information regarding the purpose of the research project and completed an informed consent form prior to the interview. Students were not provided any incentive to participate and all identifying data were removed from the interview transcripts.

## Results

Demographic information is reported in Table 1. All participants (*N* = 9) were female, a gender imbalance which is common for the nursing profession and nursing student cohorts [13]. The majority (*n* = 7) were aged over 25 (mean 29 years), which is consistent with a reported higher rate of mature-aged woman enrolled as rural nursing students [5]. Two participants identified as Aboriginal and/or Torres Strait Islander, which is also reflective of the local community, where 23% identify as Aboriginal and/or Torres Strait Islander [22] and all participating students were employed at the time of the study.

**Table 1:** Participant demographic information

Characteristic	n
Gender	
Female	9
Age, years	
21–25	2
26–30	3
31–35	4
Mode of enrolment	
Internal only	3
Mixed mode (internal and external)	6
Rurality of origin	
Australia, MM* 2–3	2
Australia, MM* 4–5	1
Australia, MM* 6–7	4
Overseas	2
Dependants	
Yes	4
No	5
Ethnicity	
European/Caucasian	6
Aboriginal and/or Torres Strait Islander	2
Other	1
Current employment status	
Part-time/Casual	8
Full-time	1
Highest level of education before enrolment	
Year 12	1
Vocational education and training	5
Undergraduate degree	1
Postgraduate degree	2
Highest Education level of parents	
Year 10	3
Year 12	2
Vocational education and training	1
Undergraduate degree	1
Postgraduate degree	2

*Modified Monash category of geographical remoteness [20]

Following analysis, the findings from the data were summarised in common themes, to achieve an understanding of the students’ experiences including remote campus experiences, learning experiences, and relationships and support. These are supported by verbatim quotes from participants below.

### Remote campus experiences

Participants described the unique experiences and opportunities they had due to their decision to study at a satellite campus located in a remote town. These included positive opportunities such as being able to achieve their aspirations of working as a nurse in their remote communities, being able to study without leaving home, and employment at the local hospital; and negative experiences such as disconnection from the university and a perceived lack of the ‘campus experience’.

Participants reflected on their past aspirations for choosing to study nursing, which included having a rewarding profession, learning further skills, a change in career, and future career opportunities. One participant also admitted that they would not have enrolled in university at all if the campus was not located in their hometown:

“I just sort of knew they offered it out here too, I thought that’s good, at least I don’t have to go away, I can stay here and work … Because I grew up here too, I think it was nice to be able to do my degree here.” (IV2)

Studying at the remote university campus was instrumental in allowing participants to meet their previous aspirations of wanting to build their careers without having to relocate. It also provided opportunities in relation to future work aspirations. One participant had the opportunity to work at the local hospital as a student nurse, which allowed her to establish and build relationships with staff and she hoped this would lead to further work in the future:

“We’ve made a lot of good connections with people who hopefully we’ll one day get to work with, or we’ve already been working with because I work at the hospital as well … it’s really good that we’ve gotten to experience that.” (IV2)

Participants reflected on how geographical remoteness affected their university experiences, participation, or satisfaction, and expressed feelings of disconnection between the remote campus and the main campus. While many of the university’s support services are offered by distance from the main campus, the modes of contact to access these services were not considered to be ideal:

“Support services, (university) does offer all their online counselling services and things like that. Harder to connect with them out here because it’s via phone and it’s not as comfortable.” (IV5)

Participants expressed feelings associated with ‘being at university’ or feelings of disconnection from the main campus, due to living and studying at a satellite campus in a remote town. Some participants stated that their feelings regarding ‘being at university’ did not meet their expectations, or were different to what they experienced during their visits to the main campus:

“You don't get that campus experience, like everybody talks about, I've never had that experience … You don't really feel like you’re at uni.” (IV7)

### Learning experiences

Participant learning experiences were impacted by a number of factors, including stress felt due to other competing priorities; the ability to engage with their classes; experiences of videoconference or recorded lectures and face-to-face tutorials; and the learning benefits of being part of a small cohort.

Participants reflected on the challenges of balancing their different roles as students, employees, and mothers, the physical stress they experienced as a result, and how these affected their study goals and experiences:

“At the start, in my first year … I really burnt out by like week six.” (IV7)

Participants felt more connected during the live videoconference lectures when teaching staff based in the main campus engaged them directly by asking questions and including them in discussions. Conversely, when the lecturer did not engage the remote campus students, they felt excluded:

“In a lot of courses, the lecturer would actually go through campuses and direct questions at specific campuses … So, that felt good, that was engaging.” (IV5)

Participants described how lectures delivered via videoconference from the main campus were more difficult to engage with, describing them as ‘dragging’ and ‘long’. They also expressed that being visible to other sites made them feel uncomfortable:

“It was just sometimes the lectures were a bit too long and it’s like, we were here and everyone’s in that classroom (at main campus) watching … .” (IV6)

Technical difficulties were often described as a barrier to engaging in videoconference lectures, as issues with the connection or hardware occurred intermittently:

“Technical difficulties. I mean, it’s something that happens all the time. But when you can’t login or the link code doesn’t work that makes it quite frustrating.” (IV5)

Participants expressed their feelings of discomfort during live videoconference lectures, particularly when they were required to interrupt the lecture to ask a question, as they were concerned that they may be perceived as being disruptive:

“No. Did not feel comfortable. It was disjointed communication.” (IV5)

One participant discussed that having lectures delivered as recordings rather than a live videoconference in the classroom affected their motivation, as it reduced their levels of engagement, made them feel isolated and affected their motivation to study. However, some participants viewed recorded lecturers positively, as they could contact their lecturer by other means following the lecture rather than having to ask their questions in front of other students:

“I feel like I can still ask questions, through email … It’s convenient because it takes that shyness of facing the teacher directly away.” (IV1)

Participants also enjoyed the flexibility that recorded lectures provided:

“Now they’re all recorded lectures, and you can play it at any time. At first, I didn’t like it, now I think that it’s more user friendly.” (IV1)

Participants enjoyed attending residential classes, particularly the chance to meet teaching staff based at the main campus as well as other students:

“They involve everyone in the discussion … It is a much bigger class, but there was enough staff to still make it feel like you’re not left alone.” (IV3)

Face-to-face delivery of tutorials was viewed positively by all participants, as they facilitated discussion between students and their tutors. They also appreciated having tutors who were currently practicing at the local hospital:

“I enjoy the fact that tutors are employed at the hospital, and so they present their experience, they support their education with their experiences, they share their activities from their work.” (IV1)

However, some participants also reflected on disruption caused to the continuity of their studies due to frequently changing, casually employed tutors prioritising their full-time jobs or shift work at the hospital:

“They’re great, face-to-face … but we’ve only had two classes where we consistently had a teacher through the entire semester … So there’s no consistency there, they don't know what was picked up from the last one.” (IV9)

Two participants expressed their preference for the small class sizes at the remote campus, as they were more comfortable during tutorials or asking questions in front of smaller groups of people:

“Because we’re such a small cohort it was easier, you weren’t walking into this big classroom … like (main campus), where there’s like 80 something people in there and trying to get things across … I felt more comfortable in those tutes.” (IV4)

During practical classes, the smaller cohort was perceived to be a particular advantage, as students had more time to participate in each individual learning activity, increasing their confidence with technical skills:

“We have a lot more time to do each individual activity. So, we get a lot more hands-on time, with each one, to be able to have a lot more practice and confidence and stuff with it.” (IV2)

However, one student reflected on the ways that a poor relationship with teaching staff or other students in their small cohort had a negative effect on her feelings of personal safety and anxiety, which in turn, affected her learning experiences:

“If I don’t get along with a tutor, this interferes with the whole experience. I don’t feel safe, if there is an interference, either with the tutor or with the other students, because of the small cohort.” (IV1)

### Relationships and support

Participants favourably described the support services and facilities available to them locally at the remote satellite campus, including support from academic, library and administrative staff, the university, the local UDRH, and access to a health library, computer laboratory, and Wi-Fi. The familiarity and relationships that participants developed with campus staff and other students were extremely important and affected their university learning experiences. The existence or lack of support from family, partners, friends, and the wider community, also had a great impact on participants' ability and motivation to study.

The local nursing lecturer was described as providing both academic and emotional support, and Aboriginal and Torres Strait Islander students could also receive additional support from the UDRH’s Head of Indigenous Health:

“I’ve had (remote campus nursing lecturer) skim over a few things and she’s helped me with my confidence in my maths calculations, because I was really panicking about those, but she was really good with those. She’d spend that extra time with me.” (IV4)

However, another student described her difficulties in forming mentor-mentee relationships due to the small number of nursing academic staff employed at the campus:

“Harder to find an academic mentor in a rural facility because we just don’t have a lot of people on campus.” (IV5)

Participants reflected on significant support strategies that were provided to them on-site such as visits from main campus staff during orientation week and during the semester. Visits from the college’s liaison librarian and lecturers based at the main campus visiting during the semester were highlights for the students:

“ … they have those academic boot camps, so they get the librarian from (main campus) to come out … I did go to one at the start, and he went over academic writing, which helped me again.” (IV7)

Students occasionally received support from administrative staff at the UDRH, including arranging IT support, and printing and binding study materials. Access to a clinical library and computer lab were particularly valuable to participants, as they had access to clinical texts, computers, and free printing facilities:

“So the fact that (library staff) proofread, and the fact that I can print for free, and I use the computer lab to do my exams.” (IV1)

Students had access to Wi-Fi at the remote campus, which was particularly appreciated by students who did not have access to internet at home:

“I used the (Wi-Fi) quite often because I didn’t have Wi-Fi at home for the first two years of my degree.” (IV5)

However, the lack of access to these facilities outside of business hours was viewed as a barrier, and was compared to the main campus where 24-hour library and computer facilities are available:

“I find it challenging that there’s no library over the weekends or after hours.” (IV1)

Both participants who identified as Aboriginal and Torres Strait Islander had access to additional Indigenous student support services from the university:

“The Indigenous Centre with the tutoring … they ring you up all the time. Check in on you. Try and give you advice. Then when we go to (main campus), we get full access to the Indigenous Centre, including after hours.” (IV8)

Interestingly, all non-Indigenous participants were aware of this tutoring service, and felt that a similar service could benefit other students at the remote satellite campus:

“It’d be good if they had something like that out here … where they get people like (student) … just to come in and help with the first years.” (IV7)

One participant was particularly frustrated by the lack of financial support provided to remote campus students when they were required to travel for clinical placements. They compared this to students from metropolitan universities who travel to undertake rural clinical placements who at times are provided with free or subsidised accommodation and travel:

“We don’t get that at all. We get nothing, which is frustrating. I think they also get something to do with their travel as well, travelling to remote areas. It annoys me.” (IV8)

They also described financial and logistical barriers faced in attending mandatory residential blocks, including having to travel over 900 km to and from the main campus, and then travelling between the university campus and accommodation in a larger city. However, some of these barriers were offset by the UDRH:

“(UDRH) personally refunded me nearly the full amount, (they) paid for accommodation, and they refunded me the flights. It was very good.” (IV1)

Participants valued the relationships they had made with other members of their cohort, as well as local staff, who were familiar to the students:

“We’re just so lucky we have the relationships we can have here. And the support from everybody, like (library staff), they always ask how we’re going, what are we up to, how’s placement.” (IV2)

Conversely, some participants described how they have reduced opportunities to socialise with other students compared to those based on the main campus:

“Socially there just isn’t the cohort available to actually engage with peers.” (IV5)

Participants also reflected on their personal relationships and how their studies were supported financially and emotionally by family, partners, and employers, including continuing to live with parents, childcare, and flexible working arrangements to accommodate class attendance and clinical placements. This was particularly important during placements and residential weeks when students were required to travel:

“It was studying, building a relationship with my daughter, because she was only one … my support systems, my family, were really important to have.” (IV8)

However, one student felt she had no family support to pursue her studies, which was a cause of disappointment for her:

“No, I had no family support here to study. I don’t even think they remember I study. I think my family being very rural minded, they don’t necessarily care for higher education … so that support is lacking.” (IV5)

Some students also noted that the local community was supportive of the nursing students, and valued the presence of the remote campus:

“I notice in the community that they know that I’m studying nursing, and so they would come to encourage me, or they ask me about what it’s like studying nursing, how it is … the community appreciates that there’s this campus.” (IV1)

## Discussion

This study aimed to explore and describe the experiences of nursing students at a remote satellite university campus, with a focus on their experiences of local and distance education methods; staff, support, and services; and barriers and enablers to successful study at a remote campus. These results have shown that there are a range of learning experiences, support issues, and barriers and enablers unique to the remote satellite campus context that differ from the experiences of nursing students studying on metropolitan campuses.

Learning experiences at regional university campuses differ from those at metropolitan universities, due to factors such as student diversity, life experiences, and class size [5, 23]. Participants’ reflections regarding a sense of ‘not feeling like you’re at uni’, highlight the importance of feeling like a part of the university institution. A study by Delaney [24] found that students studying by distance reported feeling ‘less integrated’ in the institution than full-time internal students. Social and academic integration into ‘university life’ and increased levels of support, including fostering a sense of belonging, may reduce feelings of exclusion and isolation, that are common when beginning university, particularly for first-generation students [13].

There have been concerns expressed regarding the quality and student satisfaction of education delivered by distance, including videoconference or recorded lectures due to the lack of face-to-face interaction between students and teaching staff [10, 23, 25, 26]. While the use of videoconferencing and recorded lectures have previously been viewed less positively compared to face-to-face delivery, recorded lectures have their own advantages as they allow for greater flexibility and can be viewed online when convenient [10, 23]. As the use of videoconferencing and streaming has become more mainstream, these views may have changed as participants in the current study generally viewed recorded lectures positively. Negative remarks regarding videoconference lectures generally included technical issues, which are not uncommon in regional, rural and remote Australia [27], and hesitancy to ask questions due to shyness or anxiety. This was also a common barrier to engaging in study for students attending a remote nursing campus in the Torres Strait Islands [28]. In the wake of the COVID-19 global pandemic, these findings may also be relevant across all geographical contexts and disciplines, due to the increased reliance on online services and remote technologies such as streaming recorded lectures and use of virtual classroom services to deliver course content.

Teaching staff at remote satellite university campuses are often hired from local industry [27], which was viewed positively by participants in this study, however it did cause issues with consistency of tutorial staff and was perceived to interfere with participants’ learning.

The results of this study emphasise the importance of relationships and positive interactions between rural and remote campus students and local staff. Small class sizes are common across regional and satellite campuses, and are consistently reported as a positive factor, as they facilitate close, supportive relationships with other students and enable more individual interactions between staff and students [10, 23] which was appreciated by participants in the current study. However, while student experiences of being part of a small cohort were generally positive, it could also result in tension and discomfort for students in the case of a poor relationship with another student or a staff member.

Participants reflected on feelings of disconnection from the university, based on the vast distance between the sites. Students at rural and remote satellite campuses may feel unsupported by their university, due to difficulties communicating with the main campus and lack of awareness regarding resources available for students [10, 29]. Reduced access to resources including library and computing facilities, and access to reliable internet both on campus and at home also presents a challenge for rural and remote students [10, 23, 29].

Students reported access to several support services provided by the university and the UDRH, including academic, library, career, and pastoral support. The academic and pastoral support from the remote nursing lecturer described in the current study also correlates with research by Wirihana et al., who found that nursing academics on satellite campuses felt they met the needs of students on satellite campuses by recognising student inequities, and “going the extra mile to support students” [27]. The additional support for Aboriginal and/or Torres Strait Islander students from the university including tutoring, learning advisors, and access to a physical study space while visiting the main campus can contribute to motivation and success at university [30].

Several demographic features of remote university campus cohorts are often associated with barriers to successful study and completion of undergraduate nursing programs. The demographic profile of the participants of this study correlate with literature findings that rural and remote campus nursing students are more likely to be mature-aged women, often balancing their studies with family and work demands, which may sometimes take priority over their educational obligations. They often require employment whilst studying to support themselves and their families financially. Rural university campus students are also more likely to have completed year 12 than their parents and be first generation students compared to their metropolitan counterparts [5, 23]. Rural university campuses have a higher proportion of students from low socio-economic backgrounds and culturally and linguistically diverse backgrounds such as Aboriginal and Torres Strait Islander cultures [13, 23, 28, 31]. First generation students, mature-aged women and rural and remote students are considered ‘at-risk’ students, experiencing poorer academic results and retention rates than traditional university students [23]. The physical presence of the remote university campus providing opportunities for undergraduate nursing study, combined with the financial support provided by the local University Department of Rural Health to attend residential blocks at the main campus is also an enabling factor for students [14]. Family support was mentioned by most participants as enabling them to continue with their studies, a frequent benefit for rural nursing students [5, 10, 13]. Community support and respect, as described by participants, is also valued by remote campus students, and may contribute to student motivation [10].

The notion that nursing students who attend rural and remote campuses are more likely to choose to work in these areas after graduation [6, 10, 11] is supported, as some participants were already employed at the local hospital as student nurses. Cosgrave et al. reported that a lack of social connections is among the factors that contribute to high turnover rates of rural health professionals [4]. Conversely, social connections and integration, rural familiarity, and community participation and satisfaction including a sense of belonging can contribute to rural nurses’ decision to continue employment in rural areas.

A limitation of this study is volunteer bias, as students could elect to participate and may have only done so because they were interested in the topic of the research project. Additionally, there were a small number of participants, which is typical of studies undertaken at these campuses [10, 11, 13, 23, 31]. Due to the nature of a small campus, the researcher was also known to some of the participants which may have influenced the decision to participate or withhold sharing certain experiences.

## Conclusions

Having a rural background and attending a rural university campus may positively influence nurses’ choices to live and work in rural and remote Australia. While students enrolled at a geographically remote university campus will undoubtedly have different learning experiences and face additional barriers to participation in tertiary study compared to metropolitan students, they also benefit from a range of enabling factors that are associated with their experience and their personal, community, and student relationships. An improved understanding of the remote nursing campus student experience and the barriers faced by these students may influence support services so that they are tailored to the local student population, potentially improving student outcomes and retention of these health professionals in rural and remote locations.

## Data Availability

The dataset generated and/or analysed during the current study is not publicly available due to confidentiality. It can be obtained from the corresponding author on reasonable request.

## Abbreviations

BNSc: Bachelor of Nursing Science
MM: Modified Monash Model category (20)
UDRH: University Department of Rural Health

## References

[1] Australian Institute of Health and Welfare. Rural and Remote Australians. 2019. https://www.aihw.gov.au/reports-data/population-groups/rural-remote-australians/overview. Accessed 1 Mar 2020.

[2] Collett MJ, Fraser C, Thompson SC (2020). Developing the future rural nursing workforce: report on a nursing roundtable. Collegian (Royal College of Nursing, Australia).

[3] Russell DJ, Zhao Y, Guthridge S, Ramjan M, Jones MP, Humphreys JS (2017). Patterns of resident health workforce turnover and retention in remote communities of the Northern Territory of Australia, 2013–2015. Hum Resour Health.

[4] Cosgrave C, Malatzky C, Gillespie J (2019). Social determinants of rural health workforce retention: a scoping review. Int J Environ Res Public Health.

[5] Croxon L, Maginnis C (2007). The total learning environment and implications for rural student nurse retention. FoHPE.

[6] Playford D, Wheatland B, Larson A (2010). Does teaching an entire nursing degree rurally have more workforce impact than rural placements?. Contemp Nurse.

[7] Shires L, Allen P, Cheek C, Deb W (2015). Regional universities and rural clinical schools contribute to rural medical workforce, a cohort study of 2002 to 2013 graduates. Rural Remote Health..

[8] Campbell N, Stothers K, Swain L, Cairns A, Dunsford E, Rissel C (2020). Health services in northern Australia depend on student placements post COVID-19. Aust N Z J Public Health.

[9] Fitts MS, Russell D, Mathew S, Liddle Z, Mulholland E, Comerford C (2020). Remote health service vulnerabilities and responses to the COVID-19 pandemic. Aust J Rural Health.

[10] Gum LF (2007). Studying nursing in a rural setting: are students adequately supported and prepared for rural practice? A pilot study. Rural Remote Health.

[11] Birks M, Al-Motlaq M, Mills J (2010). Pre-registration nursing degree students in rural Victoria: characteristics and career aspirations. Collegian (Royal College of Nursing, Australia)..

[12] Rossi F, Goglio V (2020). Satellite university campuses and economic development in peripheral regions. Stud Higher Educ (Dorchester-on-Thames).

[13] Christensen M, Medew K, Craft J (2019). “Nursing tree time”: an inter-professional team approach to supporting student nurse learning at a regional university campus. Nurse Educ Today.

[14] Nugent P, Ogle KR, Bethune E, Walker A, Wellman DA (2004). Undergraduate pre-registration nursing education in Australia: a longitudinal examination of enrolment and completion numbers with a focus on students from rural and remote campus locations. Rural Remote Health.

[15] Hays C, Devine S, Wongtongkam N, Glass B (2021). Studying nursing at Australian satellite university campuses: a review of teaching, learning and support. Aust J Rural Health.

[16] Ajjawi R, Higgs J (2007). Using hermeneutic phenomenology to investigate how experienced practitioners learn to communicate clinical reasoning. Qual Rep.

[17] Neubauer BE, Witkop CT, Varpio L (2019). How phenomenology can help us learn from the experiences of others. Perspect Med Educ.

[18] Van Manen M (1990). Researching lived experience: human science for an action sensitive pedagogy.

[19] Lopez KA, Willis DG (2004). Descriptive versus interpretive phenomenology: their contributions to nursing knowledge. Qual Health Res.

[20] Department of Health. Modified Monash Model. 2019. https://www.health.gov.au/health-workforce/health-workforce-classifications/modified-monash-model. Accessed 3 Sept 2019.

[21] Rich S, Graham M, Taket A, Shelley J (2013). Navigating the terrain of lived experience: the value of lifeworld Existentials for reflective analysis. Int J Qual Methods.

[22] Australian Bureau of Statistics. 2016 Census Quickstats: Mount Isa. 2018. https://quickstats.censusdata.abs.gov.au/census_services/getproduct/census/2016/quickstat/SED30057. Accessed 3 Sept 2019.

[23] Maginnis C, Croxon L (2005). Diversity in rural students : identifying student learning needs. Educ Rural Aust.

[24] Delaney L, Brown M (2018). To walk invisible: distance students in a dual-Mode University. Distance Educ.

[25] Usher K, Lindsay D, Mackay W (2005). An innovative nurse education program in the Torres Strait Islands. Nurse Educ Today.

[26] Wines M (2016). Nursing education in rural environments. Compr Psychol (Missoula, MT).

[27] Wirihana L, Welch A, Williamson M, Christensen M, Bakon S, Craft J (2017). The provision of higher education in regional areas: an integrative review of the literature. J High Educ Policy Manag.

[28] Felton-Busch C, Maza K, Ghee M, Mills F, Mills J, Hitchins M (2013). Using mentoring circles to support Aboriginal and Torres Strait islander nursing students: guidelines for sharing and learning. Contemp Nurse.

[29] Ostini J, Partridge H, Kelly K, Owen S, Jeffries S (2020). Narratives of access: a critical exploration of how institutional interactions with students affect regional student participation in higher education. Student Success.

[30] Oliver R, Grote E, Rochecouste J, Dann T (2016). Indigenous student perspectives on support and impediments at university. Aust J Indigen Educ.

[31] Penman J, White F (2006). Peer-mentoring program 'pop-up' model for regional nursing students. J Univ Teach Learn Pract.

